# Development of PCR‐Based Markers to Determine the Sex of Kelps

**DOI:** 10.1371/journal.pone.0140535

**Published:** 2015-10-23

**Authors:** Agnieszka P. Lipinska, Sophia Ahmed, Akira F. Peters, Sylvain Faugeron, J. Mark Cock, Susana M. Coelho

**Affiliations:** 1 Sorbonne Université, UPMC Univ Paris 06, Algal Genetics Group, UMR 8227, Integrative Biology of Marine Models, Station Biologique de Roscoff, CS 90074, F-29688, Roscoff, France; 2 CNRS, Algal Genetics Group, UMR 8227, Integrative Biology of Marine Models, Station Biologique de Roscoff, CS 90074, F-29688, Roscoff, France; 3 Bezhin Rosko, 29250, Santec, France; 4 Sorbonne Universités, UPMC University Paris 06, UMI 3614, Evolutionary Biology and Ecology of Algae, Station Biologique de Roscoff, CS 90074, F-29688, Roscoff, France; 5 CNRS, Evolutionary Biology and Ecology of Algae, Station Biologique de Roscoff, CS 90074, F-29688, Roscoff, France; 6 Centro de Conservación Marina and CeBiB, Facultad de Ciencias Biológicas, Pontificia Universidad Católica de Chile, Santiago, Chile; CNRS, FRANCE

## Abstract

Sex discriminating genetic markers are commonly used to facilitate breeding programs in economically and ecologically important animal and plant species. However, despite their considerable economic and ecological value, the development of sex markers for kelp species has been very limited. In this study, we used the recently described sequence of the sex determining region (SDR) of the brown algal model *Ectocarpus* to develop novel DNA-based sex-markers for three commercially relevant kelps: *Laminaria digitata*, *Undaria pinnatifida* and *Macrocystis pyrifera*. Markers were designed within nine protein coding genes of *Ectocarpus* male and female (U/V) sex chromosomes and tested on gametophytes of the three kelp species. Seven primer pairs corresponding to three loci in the *Ectocarpus* SDR amplified sex-specific bands in the three kelp species, yielding at least one male and one female marker for each species. Our work has generated the first male sex-specific markers for *L*. *digitata* and *U*. *pinnatifida*, as well as the first sex markers developed for the genus *Macrocystis*. The markers and methodology presented here will not only facilitate seaweed breeding programs but also represent useful tools for population and demography studies and provide a means to investigate the evolution of sex determination across this largely understudied eukaryotic group.

## Introduction

Brown algae (Phaeophyceae) are the dominant inhabitants of coastal environments and the major primary producers in these marine ecosystems. They are best known for forming kelp forests in shallow, rocky, cold water environments, especially in the northern Hemisphere. Subtidal kelp forests, together with their associated fauna and flora, are among the most diverse and productive ecosystems worldwide (reviewed in [1–3]). The importance of brown algae as a support for ocean biodiversity and for human economic activities such as fisheries is defined directly by their role in providing food and a structural habitat in these ecosystems (reviewed in [4–6]). Brown algae are also of considerable commercial value in themselves. The seaweed farming business is growing at a rate of 7.5% every year [7], providing resources for various sectors of industry. In addition to being a staple item of the Asian diet, algae are exploited for biomedical, pharmaceutical and cosmetic applications, as nutritional supplements, as feed for animal aquaculture and for bio-energy development [8–10] reviewed in [4,11]. According to the 2014 FAO report, worldwide harvest of seaweeds more than doubled over the past 15 years and has reached 23.8 million tons (wet weight) per year, with over 96% of the global algae harvest coming from aquaculture.

Some of the most important seaweed genera, from both ecological and economical perspectives, belong to the order Laminariales (commonly referred to as kelps). This order consists of nine families and 130 species (Algaebase, accessed on the 4^th^ February 2015) [12]. *Saccharina japonica* and *Undaria pinnatifida* are the most intensely harvested brown algal species, with their production being principally located in Asia. These two species alone account for over 30% of the recent growth in the seaweed industry (FAO, 2014). Pilot aquaculture programs have also been initiated in Chile for the production of alginates and abalone feedstock from the giant kelp *Macrocystis pyrifera* [13]. The production strategies of these *M*. *pyrifera* cultivation programs are being constantly improved [14–16] but commercial output is impacted by complex morphological and reproductive variations within the cultivated populations [17].

**Fig 1:**
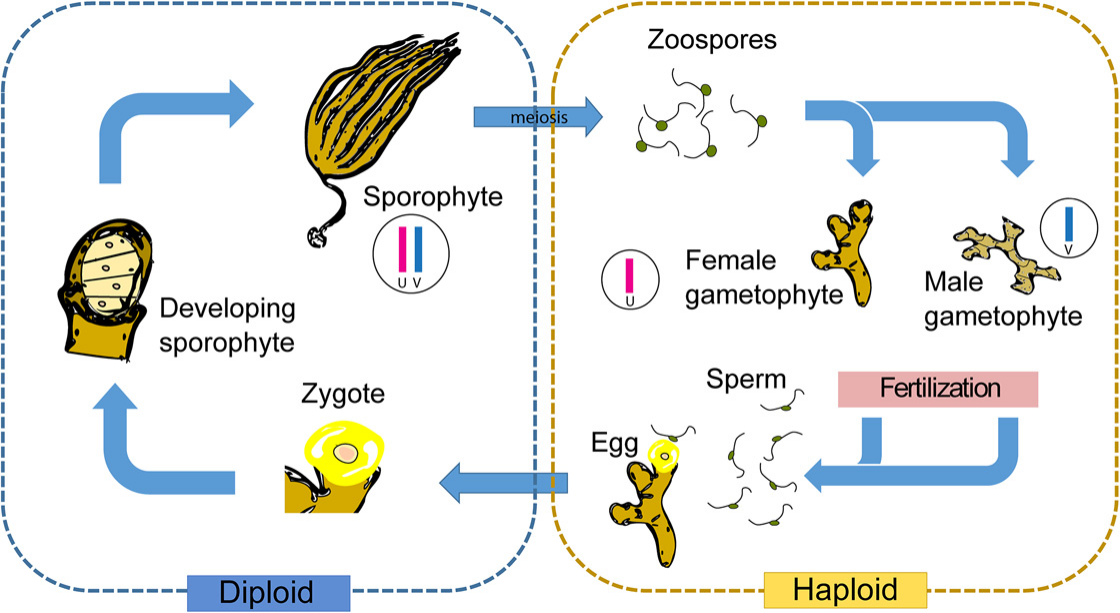
Schematic representation of a kelp life cycle. The sexual cycle consists of an alternation between a macroscopic diploid sporophyte and microscopic, haploid, dioicous (male and female) gametophytes. The zoospores produced by the sporophyte are released and develop as male (carrying the V sex chromosome) or female (carrying the U sex chromosome) gametophytes. Egg and sperm produced by the gametophyte fuse to return to the diploid, asexual sporophyte generation, which carries both the U and the V sex chromosomes.

The haploid-diploid life cycles of kelps involve alternation between a macroscopic sporophyte and microscopic, filamentous gametophyte generations, with male gametophytes having generally smaller cells than females [18] (Fig 1). In modern nurseries gametophytes and zygotes are generated under controlled laboratory conditions in order to produce the young sporophytes that are attached to cultivation ropes subsequently transferred to the open sea [19,20]. This process allows the integration of genetically defined material into the production process and has led to the emergence of seaweed breeding programs. In China, improved *Saccharina* cultivars have been produced by hybridizing gametophytes of *S*. *longissimia* and *S*. *japonica* [21,22]. These hybrids exhibit substantially increased production yield and are better adapted to a range of culture conditions. Breeding strategies have also been employed to optimise *M*. *pyrifera* cultivars [23]. In particular, experiments using crosses between *M*. *pyrifera* individuals from different geographical locations in Chile indicated applicability of heterosis breeding. Outbreeding enhancement techniques are extensively used in plant and animal breeding. In *M*. *pyrifera* and *S*. *japonica*, production of genetically homogeneous parent lines for this purpose is fairly easy, since free-living gametophytes can be propagated vegetatively and can be stored without affecting their stability or fertility over decades [23]. However, compared with the advances that have been made with terrestrial plant crops, for example, these breeding programs are still in their infancy, and there remains considerable scope for improvement of seaweed cultivars.

One of the factors limiting genetic improvement of brown seaweeds is our poor understanding of many aspects of the biology of these organisms. As members of the Stramenopiles, brown algae are very distantly related to well-studied lineages such as land plants and animals [24]. The large evolutionary distance that separates brown algae from these lineages means not only that the former possess many novel developmental, physiological and metabolic features but also that model systems developed for the other lineages are of limited relevance for studying and characterising these novel features. Over the last decade, however, the filamentous alga *Ectocarpus* has emerged as a model system for the brown algae and is being used to investigate the genetic basis of many aspects of brown algal biology [25–30]. A number of genetic and genomic resources are now available for *Ectocarpus*, including a complete genome sequence [31,32], a genetic map [33], gene mapping techniques, a large amount of transcriptomic data [34–37] and bioinformatics tools [38,39].

In this study, our objective was to use recently obtained knowledge about the sex-determination system in *Ectocarpus* to develop sex markers for commercially important kelp species. Genetic sex markers have broad applications in land plants such as jojoba [40], asparagus [41], papaya [42], ginkgo [43], hop [44], pistachio [45], and in animals such as chicken and fish produced by aquaculture [46–49]. For brown algae, genetic sex markers would represent an important tool for the efficient identification of male, female or bisexual individuals in breeding and production programs. Because gametophytes are small and isolation of single gametophyte clones is laborious, sex markers would allow rapid detection of contamination when only one haploid male or female clone is necessary. Moreover, sex markers are also useful to discriminate between diploid sporophytes derived from sexual reproduction and female or male partheno-sporophytes which can be diploid but fully homozygous. A method has recently been developed in which partheno-sporophytes are derived from female *Undaria* gametophytes. The zoospores produced by one of these partheno-sporophytes develop as gametophytes producing a large population of clonal, sexual individuals that can be used in crosses to produce large numbers of highly similar sporophytes [50]. Efforts are currently being made to develop reproductive male partheno-sporophytes for the same purpose. Mass crosses between sets of genetically identical gametophytes produced from zoospores would greatly improve breeding strategies for kelps and sex markers would represent a valuable resource for this approach, allowing verification of the unisexual genetic background of the parent partheno-sporophytes.

So far, very few sex markers have been developed for commercially grown seaweeds, rare exceptions including male and female markers for the red algae *Gracilaria gracilis* [51] and *G*. *chilensis* [52] and female microsatellite-based markers for the brown algae *S*. *japonica* [53,54] and *U*. *pinnatifida* [55]. Note, however, that when only female markers are available, it is not possible to distinguish male samples from female samples for which the PCR amplification has failed. Programs that use female markers alone therefore run the risk of a high level of falsely assigned males. Moreover, on a more general level, microsatellite-based markers have relatively high development costs, technical challenges with regards to the construction of libraries and primers, and the fact that flanking sequences may not be conserved across different groups of organisms. These features can make it difficult to transfer microsatellite-based markers across species. In addition, SDRs have not been precisely mapped in kelp species and it is possible that microsatellite loci identified as sex markers lie very close to, but not within, the SDR. If this is the case the markers may recombine with the SDR in rare instances resulting in false sex assignment. The best way to avoid this problem is to design sex markers based directly on the SDR region.

In *Ectocarpus*, sex is determined genetically during the haploid, gametophyte generation by a UV sex chromosome system [26]. The female and male haplotypes of the sex-determination region (SDR) each extend over about one Mbp and constitute about a fifth of the length of the sex chromosome (the remaining four fifths corresponding to the recombining pseudoautosomal region, the PAR, [56]). The male and female haplotypes of the SDR are thought to have stopped recombining at least 70 Mya ago [26] and are highly divergent at the sequence level. One consequence of this sequence divergence is that sex markers can easily be generated based on the amplification of either male or female sequences from within the SDR [26]. Moreover, because the divergence of the male and female SDR haplotype is relatively ancient, these regions can also be used to develop sex markers for other brown algal species, provided they share the same sex-determination system. Chromosome staining experiments and localization of the female specific SCAR marker strongly suggested that one of the chromosomes in *Saccharina japonica* carries a female-specific region[57] which was recently confirmed by a high density SNP linkage map [58]. However, a male-specific locus could not be visualized due to lack of male-specific markers.

Here we show that sequences from within the male or female SDR can be used to develop sex markers for diverse species of economically important kelps. We present the first report of male sex-specific markers for *Laminaria digitata* and *Undaria pinnatifida*, as well as the first sex markers developed for the genus *Macrocystis*. Our findings are expected not only to facilitate breeding programs of these seaweeds, but also to accelerate the study of kelps at the population and biogeographic level and to enable further research into the evolution of sex determination systems across different brown algal lineages.

## Materials and Methods

### Biological material and DNA extraction

Diploid sporophytes of *L*. *digitata*, *U*. *pinnatifida* and *M*. *pyrifera* were collected in the North Atlantic, Mediterranean, and Southeast Pacific (Table 1). Gametophytes were obtained from released zoospores maintained in culture in Petri dishes at 13°C in autoclaved natural sea water (NSW) supplemented with half-strength Provasoli solution [59] with a light:dark cycle of 12h:12h (initially, to avoid gametogenesis, 1–2 μmol photons m^−2^ s^−1^, in later cultures containing larger thalli 20 μmol photons m^−2^ s^−1^) using daylight-type fluorescent tubes. Individual gametophyte clones were sub-isolated and their phenotypic sexes were determined morphologically by inspecting cell size, female individuals having significantly larger cells than males [18]. Total genomic DNA was extracted from 10–20 mg of wet tissue of using the NucleoSpin Plant II DNA extraction kit from Machery-Nagel (http://www.mn-net.com/), following the manufacturer's instructions. DNA samples were diluted to 1 ng/μl final concentration and stored at -20°C.

**Table 1:** Origins of the kelp gametophyte clones used in this study.

Species	Strain	Sex	Locality	Country	Isolated	Date
*Laminaria digitata*	CCAP1321/1	f	Helgoland	Germany	Klaus Lüning	1974
*Laminaria digitata*	CCAP1321/2	m	Helgoland	Germany	Klaus Lüning	1974
*Laminaria digitata*	Ldig KI2f	f	Kiel Bight	Germany	AFP	1994
*Laminaria digitata*	Ldig KI2m	m	Kiel Bight	Germany	AFP	1994
*Laminaria digitata*	Ldig MEf	f	Schoodic Point, Maine	USA	AFP	1997
*Laminaria digitata*	Ldig MEm	m	Schoodic Point, Maine	USA	AFP	1997
*Laminaria digitata*	LDspR(1–6)	mf*	Roscoff	France	A.Lipinska	2014
*Undaria pinnatifida*	Upin ETf	f	Bouzigues, Etang de Thau	France	AFP	1991
*Undaria pinnatifida*	Upin ETm	m	Bouzigues, Etang de Thau	France	AFP	1991
*Undaria pinnatifida*	Upin BR04A1f	f	Brest	France	AFP	2004
*Undaria pinnatifida*	Upin BR04A2m	m	Brest	France	AFP	2004
*Undaria pinnatifida*	Upin PLY09-1f	f	Plymouth	England	AFP	2009
*Undaria pinnatifida*	Upin PLY09-1m	m	Plymouth	England	AFP	2009
*Undaria pinnatifida*	Upin 3	(f)**	Saint-Malo	France	L. Lèvéque	2015
*Undaria pinnatifida*	MeI 01	(mf)*	Merdouze	France	L. Lèvéque	2015
*Macrocystis pyrifera*	ALG5 A4	m	Algarrobo	Chile	G. Montecinos	2012
*Macrocystis pyrifera*	P20	m	Pargua	Chile	C. Camus	2012
*Macrocystis pyrifera*	PUCA11 B4	m	Pucatrihue	Chile	G. Montecinos	2012
*Macrocystis pyrifera*	PUCA11 B1	f	Pucatrihue	Chile	G. Montecinos	2012
*Macrocystis pyrifera*	PUCA26 A2	f	Pucatrihue	Chile	G. Montecinos	2012
*Macrocystis pyrifera*	ALG5 A1	f	Algarrobo	Chile	G. Montecinos	2012

*Asexual sporophyte, carrying both male and female sex chromosomes

**Partheno-sporophyte derived from female gametophyte (through parthenogenetic development of unfertilized eggs)

### Marker design and PCR amplification

Sex-specific markers were designed based on the sequence of the *Ectocarpus* sp. sex chromosomes [26,32] but also took into account, where possible, similarities between *Ectocarpus* gene sequences and publically available transcriptomic data for *Saccharina japonica* [60]. Several genes located either in the SDR or in the pseudoautosomal region (PAR) were targeted (for the list of primers and genes see Table 2). Primers were designed using Primer3 [61,62]. In total, we tested 22 primer pairs corresponding to nine *Ectocarpus* SDR genes and 15 primer pairs corresponding to eight *Ectocarpus* PAR genes. Test amplifications to detect sex markers were carried out on three to seven individuals of each sex for each of the three species. PCR was carried out in 12 μL total volume containing 1 ng of genomic DNA template, 2.5 mM MgCl2, 2 mM of each dNTP, 0.5 mM of forward and reverse primer and 0.3 U Taq polymerase (GoTaq, Promega). All loci were amplified using a touch-down PCR procedure with initial denaturation for 3min at 95°C followed by 10 cycles of denaturation at 95°C for 30s, 30s annealing at X°C decreasing 1°C per cycle and elongation at 72°C for 30-90s (see Table 2 for the exact elongation time and initial annealing temperature) and then 25 cycles of denaturation at 95°C for 30s, 30s annealing at (X-10)°C and elongation at 72°C for 30-90s, with a final elongation step of 10 min at 72°C. The PCR products were separated by electrophoresis on 1.8% agarose gels stained with ethidium bromide and a 200 bp marker (Eurogentec) was used as a size reference. Amplicons were sequenced using ABI 3130xl capillary sequencer.

**Table 2:** Primer information and PCR conditions for the sex-specific marker tests carried out on the three Laminariales species *L*. *digitata*, *M*. *pyrifera* and *U*. *pinnatifida*.

Species	Marker name	Genbank accession number	Corresponding *Ectocarpus* gene	Primers		PCR product size (bp)**		PCR condition*
				Forward	Reverse	Male	Female	
*Laminariadigitata*	M_68_16_1	KP994178	Esi0068_0016 (SDR)	GTGGCCTTCTCTTCGTAGGT	TCGTCAAGGATAAGCGACCA	250	NA	D 30s, A 30s, E 30s; T_A_ = 60 -> 50 (-1deg/cycle)
	M_68_58_3	KP994179	Esi0068_0058/FeV4scaf01_4 (SDR)	GCCTCAACACACTCCTTGG	CACCAGATCCATCGTCCCAC	NA	1500	D 30s, A 30s, E 60s; T_A_ = 65 -> 55 (-1deg/cycle)
	M_248_8_1	-	Esi0248_0008 (PAR)	AAGATGCTGTCCACCACCTT	GTGCTGATCGTGGTGAACC	200	200	D 30s, A 30s, E 30s; T_A_ = 65 -> 55 (-1deg/cycle)
*Undariapinnatifida*	M_68_16_2	KP994175	Esi0068_0016 (SDR)	CATGGAAAACACAGGCCCTC	CCATCTTCCACACCTTCCCT	350	NA	D 30s, A 30s, E 30s; T_A_ = 65 -> 55 (-1deg/cycle)
	M_68_58_1	KP994176	Esi0068_0058/FeV4scaf01_4 (SDR)	TCCCTATGCAAGACTCGAGC	CTGGTTGCAGATCTCGCTTG	980	NA	D 30s, A 30s, E 60s; T_A_ = 65 -> 55 (-1deg/cycle)
	M_68_58_2	KP994177	Esi0068_0058/FeV4scaf01_4 (SDR)	GTTGGTATAACGGCGTTGGA	CACCTCCTTAAAGTTGCGGC	NA	400	D 30s, A 30s, E 30s; T_A_ = 65 -> 55 (-1deg/cycle)
	M_285_20_2	KP994174	Esi0285_0020/FeV4scaf08_1 (SDR)	CAGCTTGGAAGTGCACATGA	GGGCTGCAAACATTGATCCA	NA	600	D 30s, A 30s, E 40s; T_A_ = 65 -> 55 (-1deg/cycle)
	M_285_26_1	-	Esi0285_0026 (PAR)	GATGAGTACACCGGAGCAGT	GGGCTTCAATATCACCTGCG	500	500	D 30s, A 30s, E 30s; T_A_ = 65 -> 55 (-1deg/cycle)
*Macrocystispyrifera*	M_68_58_1	KP994181 KP994182	Esi0068_0058/FeV4scaf01_4 (SDR)	TCCCTATGCAAGACTCGAGC	CTGGTTGCAGATCTCGCTTG	800	3000	D 30s, A 30s, E 120s; T_A_ = 65 -> 55 (-1deg/cycle)
	M_68_58_2	KP994183	Esi0068_0058/FeV4scaf01_4 (SDR)	GTTGGTATAACGGCGTTGGA	CACCTCCTTAAAGTTGCGGC	NA	350	D 30s, A 30s, E 30s; T_A_ = 65 -> 55 (-1deg/cycle)
	M_285_20_1	KP994180	Esi0285_0020/FeV4scaf08_1 (SDR)	GTGGCCATCTATTCTGCTGG	CGTTCTGCCTCCGGTCGA	NA	800	D 30s, A 30s, E 60s; T_A_ = 62 -> 52 (-1deg/cycle)
	M_357_3_1	-	Esi0357_0003 (PAR)	GCCTCAACACACTCCTTGG	CGTTCTTGTCGTCCTTGTCC	900	900	D 30s, A 30s, E 60s; T_A_ = 62 -> 52 (-1deg/cycle)

*D–denaturation time, A–annealing time, E–elongation time, TA- annealing temperature.

**NA–no amplification

## Results

### Development of sex markers for kelps based on the sequence of the *Ectocarpus* sex chromosome

The objective of this study was to explore the possibility of using information from the recently characterised sex chromosome of the filamentous brown alga *Ectocarpus* to develop sex markers for economically important kelp species. Previous work has shown that the marked divergence between the male and female haplotypes of the *Ectocarpus* SDR can be exploited to develop easily scorable, PCR-based sex markers for different species within the genus *Ectocarpus* [26]. The aim here was to determine whether this approach could be extended to more distantly related brown algal taxa.

Current estimates indicate that the Ectocarpales and the Laminariales diverged about 100 Mya [63]. The search for markers therefore focused on protein-coding genes, because these sequences are more likely to show strong conservation across this evolutionary distance than other regions of the genome. Nine genes from within the *Ectocarpus* SDR were selected as candidates for marker development, but we also included eight genes from the two surrounding, recombining regions (PAR), as controls.

The *Ectocarpus* SDR includes both sex-specific (male- or female-specific) genes and genes which are present in both the U and V SDR haplotypes (so-called gametologues). Both types of sequence could potentially be exploited for the generation of sex markers because the male and female copies of gametologues are significantly different at the sequence level and markers designed from gametologue sequences have been successfully used to discriminate between sexes in different *Ectocarpus* species [26]. The nine SDR genes that were selected for marker development therefore included four sex-specific genes (two male- and two female-specific) and five gametologues (S1 Table).

Between one and five primer pairs were designed for each gene and all primer pairs were tested against at least three male and three female individuals of each of three target kelp species, with additional primer combinations being tested for some genes by combining primers from different pairs. The markers were named according to the *Ectocarpus* gene that had been used to design them, for example M_68_58_1 corresponded to marker 1 based on the male SDR gene Esi0068_0058.

**Fig 2:**
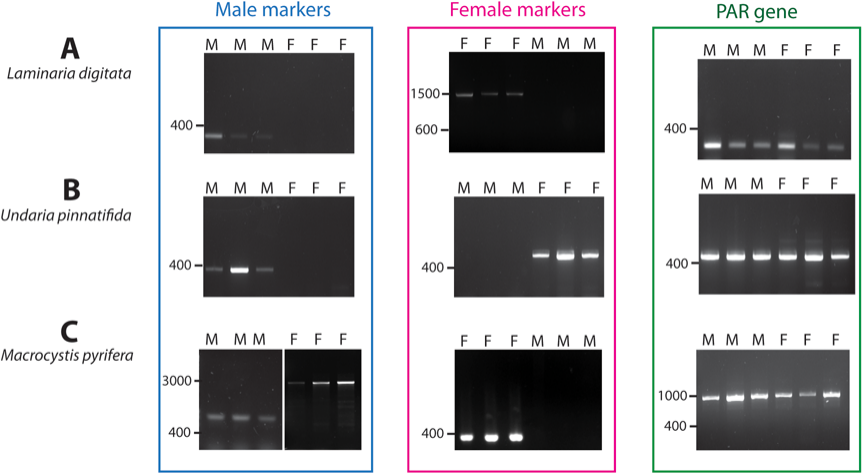
Electrophoresis pattern of products amplified in male and female gametophytes of three kelp species. (A) *L*. *digitata*. Male marker: M_68_16_1; Female marker: M_68_58_3; PAR gene amplification M_248_8_1 (B) *U*. *pinnatifida*. Male marker: M_68_16_2; Female marker: M_285_20_2; PAR gene amplification M_285_26_1 (C) M. *pyrifera*. Male marker: M_68_58_1; Female marker: M_68_58_2; PAR gene amplification M_357_3_1. M, male gametophyte; F, female gametophyte. Note that in *M*. *pyrifera*, M_68_58_1 amplified products of different sizes from male and female *M*. *pyrifera* individuals.

For the test PCR amplifications, we focused on three economically important species of kelp, *L*. *digitata*, *U*. *pinnatifida* and *M*. *pyrifera*, which represent two major families (Laminariaceae for *L*. *digitata* and *M*. *pyrifera* and Alariaceae for *U*. *pinnatifida*). PCR amplifications were performed using genomic DNA extracted from gametophytes derived from individuals that had been collected from wild populations. Of the 22 SDR gene primer pairs tested, fifteen either failed to amplify a PCR product from DNA of any of the kelp species or gave spurious amplifications. Seven primer pairs allowed the successful amplification of products corresponding to three loci (one male-specific gene, Esi0068_0016, and two gametologue pairs, Esi0068_0058/FeV4scaf01_4 and Esi0285_0020/FeV4scaf08_1; Fig 2). Primers based on the *Ectocarpus* male-specific gene Esi0068_0016 specifically amplified products from *L*. *digitata* and *U*. *pinnatifida* males. In most cases a similar pattern was observed with primers based on gametologue pairs, with a product being amplified from only one of the sexes (*L*. *digitata* females for Esi0068_0058, *U*. *pinnatifida* females for Esi0285_0020). An interesting exception was marker M_68_58_1, which amplified products of different sizes from male and female *M*. *pyrifera* individuals (Fig 2). This marker alone is therefore sufficient to determine the sex of *M*. *pyrifera* individuals but the sex-specific markers also represent valuable sex-markers provided that male- and female-specific markers are combined to avoid the occurrence of false negatives due to non-amplification. Note that, by combining markers for more than one SDR gene, it was possible to define pairs of markers that positively detected both male and female individuals for each of the three test species (Fig 2).

Comparison of the sequences of the sex-specific PCR products amplified from the kelp genomic DNAs with the *Ectocarpus* genome using Blastn consistently detected the *Ectocarpus* reference gene used for the primer design as the best match (E value 10e-04 – 5e-104).

**Fig 3:**
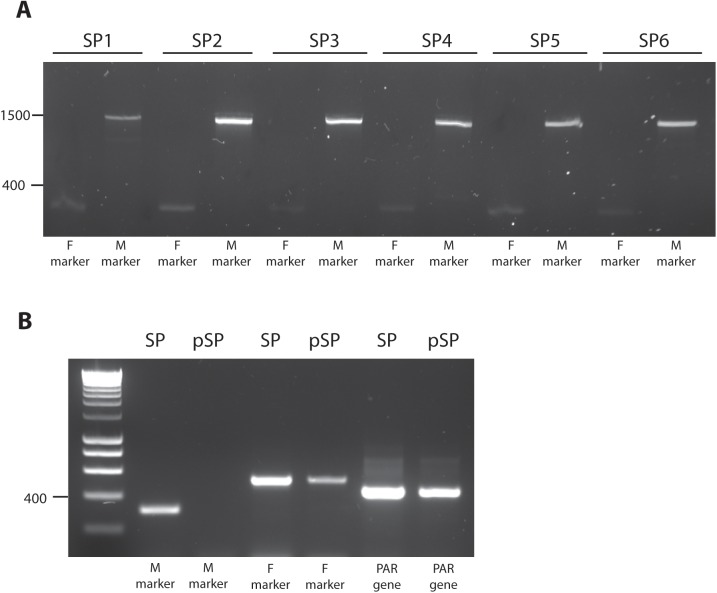
A) Electrophoresis pattern of sex marker products amplified in six diploid sporophytes of *L*. *digitata*. M: male marker (M_68_16_1); F: female marker (M_68_58_3). B) Electrophoresis pattern of sex marker products amplified in a diploid sporophyte and female partheno-sporophyte of *Undaria pinnatifida*. M: male marker (M_68_16_2), F: female marker (M_285_20_2), PAR gene (M_285_26_1); SP: sporophyte, pSP: partheno-sporophyte.

The diploid sporophyte generation is expected to carry both the male and female haplotypes of the sex locus. To determine whether male and female markers could be employed as co-dominant markers in this diploid context, six *L*. *digitata* sporophytes collected from a wild population in Roscoff, France were tested for amplification. Fig 3A shows that both the male and female markers scored positive for all six individuals. In addition, we tested the ability of the markers to distinguish between diploid sporophytes (resulting from fertilization) and partheno-sporophytes (derived from unfertilized female eggs). Fig 3B demonstrates that female partheno-sporophytes score positive only for the female marker, whereas diploid sporophytes show amplification of both male and female markers.

Of the 15 PAR gene primer pairs, 12 failed to amplify a product from DNA of any of the kelp species. We therefore retained three markers corresponding to three PAR genes for further analysis (Esi0357_0003, Esi0248_0008 and Esi0285_0026). The products amplified from males and females were of identical size suggesting that these regions are not polymorphic and recombine between sexes, and therefore can be used as PCR controls in the kelp species analysed (Fig 2).

## Discussion

### Genetic sex markers represent an important new tool for kelp breeding programs

*In vitro* production of zygotes and young sporophyte seedlings through hybridization of male and female gametophyte lines is an important process in commercial kelp cultivation [64]. The filamentous, microscopic gametophyte generation can be easily maintained in the laboratory and is used not only for controlled breeding but also for germplasm preservation and vegetative propagation [65–69]. Gametophytes can be sexed based on cell size and the presence of distinctive reproductive structures (oogonia and antheridia) but these structures appear only after several weeks of cultivation [70]. Attempts have been made to develop alternative approaches based on sexual dimorphisms to determine sex at an earlier stage of development. For example, measurement of filament width has been used, exploiting sex-dependent differences gametophyte cell size [71,72]. However, this approach cannot be applied in the species where gametophytes are monomorphic such as *Phyllariopsis brevipes* (C. Agardh) [73], which belongs to the Tilopteridales [12] but resembles a small kelp morphologically, and *Laminaria longipes* Bory de Saint-Vincent [74], for example. Another possible method is based on differences in fluorescence between the meiospores that will give rise to male and female gametophytes. Flow cytometry experiments detected lower levels of blue fluorescence in male meiospores of three Laminariales species (*Alaria marginata*, *Laminaria saccharina* and *Cymathere triplicata*) [72]. However, survival of meiospores after sorting was low (approximately 1.5%) making this an inefficient method of sex determination. Genetic sex markers represent a rapid, efficient and cost effective method to sex gametophytes and they do not suffer from the limitations encountered with the phenotypic markers described above. In particular, genetic sex markers can be reliably applied to material from any stage of the life cycle and the individuals tested do not need to be sexually mature.

Another life cycle feature that is of interest for kelp breeding is the production of sporophytes by parthenogenetic development of gametes. The homozygotic nature of these individuals allows direct selection of optimal genetic variations underlying desired phenotypic characteristics. Parthenogenesis has been observed under laboratory conditions in *S*. *japonica*, *L*. *digitata*, *M*. *pyrifera*, *U*. *pinnatifida* and *Lessonia nigrescens* when male and female gametophytes have been cultivated separately [50,75–77]. In these studies, female partheno-sporophytes were able to produce normal gametophytes that could be crossed with male gametophytes to produce diploid sporophytes [76]. The co-dominant nature of the sex-markers developed in this study allows their use to discriminate between diploid sporophytes and haploid partheno-sporophytes, and therefore informs on the success of a breeding experiment.

Taken together, our experiments indicate that *Ectocarpus* SDR sequences can be used to rapidly develop effective and broadly applicable sex markers for kelp species and possibly for other brown algae, particularly species that are more closely related to *Ectocarpus* than kelps. The wide applicability of this approach is underlined by the fact that two of the markers (M_68_58_1 and M_68_58_2) (Table 2) amplified sex-specific products from individuals corresponding to two different families within the Laminariales (Laminariaceae and Alariaceae) (Fig 1) [78,79].

### Additional applications for sex markers in population and demographic studies

The sex markers developed in this study also have multiple applications for the analysis of the structure of wild kelp populations, allowing the detection and analysis of the life cycle variations in the field. For example, it will be possible to test the degree of parthenogenetic reproduction in marginal habitats at species range limits, a phenomenon that was recently described in populations of *L*. *digitata* in Brittany [80]. The study of selection against hybrids during speciation is another possible application, as in the case of the *Lessonia nigrescens* species complex for which changes in sex ratio was observed specifically in the populations where sister-species were in close contact [81]. The availability of sex markers could allow a better understanding of the meiotic drive leading to sex ratio changes and to hybrid avoidance, and ultimately to mating system evolution. Moreover, as most of the markers span intron regions and are applicable to a broad range of strains or species, they could also potentially be used to sample polymorphism within populations by coupling PCR amplification with sequencing of PCR products.

### Sex chromosome evolution

The analyses carried out in this study provide the first evidence that sex chromosomes in two major brown algal orders, the Ectocarpales and the Laminariales, are derived from a common ancestral sex-determination system. All of the kelp sequences that were amplified using primers based on *Ectocarpus* SDR genes were sex-linked in the kelp species, whereas the markers based on PAR genes that were tested failed to detect evidence of sex-specific alleles in kelps. This suggests that the SDR of Laminariales and Ectocarpales have shared ancestry, i.e, the sex-determination system originated more than 100 Mya, before the divergence of the two orders [63]. However, more detailed information about kelp genomes, including good quality whole genome assemblies, will be required to further investigate the kelp sex chromosome structure in detail. If sex chromosome systems are conserved across major groups within the brown algae, as this study suggests, this will provide a highly interesting system to investigate how sex chromosomes evolution is influenced by modifications to the sexual system. Brown algal sexual systems are particularly diverse with regard to a number of factors, including for example relative gamete size (isogamy, anisogamy and oogamy), sexual identity (dioicy and monoicy) and the extent to which sexual reproduction is part of the life cycle (asexual cycles, parthenogenesis) [82], and it will be of considerable interest to determine how this diversity has impacted on sex chromosome evolution and function.

## Conclusion

In this study we have developed novel DNA-based sex-markers for the three economically important kelp species, *L*. *digitata*, *U*. *pinnatifida* and *M*. *pyrifera*. The markers are robust and easy to apply, because sex is determined simply by PCR-amplification of sex-specific DNA, visualized as bands on agarose gels. This is an advantage over alternatives such as microsatellite-based markers for example, which require the application of more sophisticated analyses to measure the exact size of the amplified product. Moreover, the three target kelp species were phylogenetically diverse, suggesting that the approach should be adaptable to a broad range of species within the Laminariales. The sex markers described here, and the general approach of developing sex markers based on Ectocarpus sex chromosome sequences, represent important new tools for seaweed breeding and biotechnology. They also have potential applications in natural population studies and in studies that aim at understanding the mechanisms and evolutionary principals underlying sex determination in brown algae.

## Supporting Information

S1 Table Expression levels in *Ectocarpus* and the functions of the genes used in this study. (DOCX)
