# Supplementary material for: Development of PCR‐Based Markers to Determine the Sex of Kelps
Source: PLoS One. 2015 Oct 23;10(10):e0140535. doi: 10.1371/journal.pone.0140535 (PMC4619726; doi:10.1371/journal.pone.0140535)
Supplement: S1 Table — (DOCX) [file pone.0140535.s001.docx]

| ***Ectocarpus* gene** | **Gene name** | **Average expression (RPKM)*** | | |
| --- | --- | --- | --- | --- |
|  |  | **Sporophyte** | **Gametophyte**  **male** | **Gametophyte**  **female** |
| Esi0068_0016  (SDR) | High mobility group domain protein | 4.1 | 14.0 | 0 |
| Esi0068_0058  (SDR) | STE20-like serine/threonine kinase | 13.6 | 43.8 | 0 |
| FeV4scaf01_4  (SDR) | STE20-like serine/threonine kinase | 2.0 | 0 | 51.5 |
| Esi0285_0020  (SDR) | RING-type Zinc finger domain protein | 15.2 | 23.0 | 0 |
| FeV4scaf08_1  (SDR) | RING-type Zinc finger domain protein | 16.5 | 0 | 12.9 |
| Esi0248_0008  (PAR) | expressed unknown protein | 13.2 | 3.2 | 5.1 |
| Esi0285_0026  (PAR) | PBipA, plastid GTPase BipA/TypA | 30.6 | 69.7 | 67.87 |
| Esi0357_0003  (PAR) | Inosine triphosphate pyrophosphatase, putative | 3.4 | 8.5 | 8.6 |

**S1 Table.** Expression levels in *Ectocarpus* and the functions of the genes used in this study. *expression values (Reads Per Kilobase per Million mapped reads, RPKM) were obtained from Lipinska et al. 2015.
